# Treatment of community-onset pneumonia in neutropenic cancer patients: β-lactam monotherapy versus combination antibiotic regimens

**DOI:** 10.1186/s41479-019-0061-1

**Published:** 2019-06-05

**Authors:** Hyeri Seok, Jae-Hoon Ko, Kyong Ran Peck, Ji-Yeon Kim, Ji Hye Lee, Ga Eun Park, Sun Young Cho, Cheol-In Kang, Nam Yong Lee, Doo Ryeon Chung

**Affiliations:** 1 Division of Infectious Diseases, Department of Medicine, Korea University Ansan Hospital, Ansan, Gyeonggi-do Republic of Korea; 2 Division of Infectious Diseases, Department of Medicine, Samsung Medical Center, Sungkyunkwan University School of Medicine, 81 Irwon-ro, Gangnam-gu, Seoul, 06351 Republic of Korea; 3 Department of Laboratory Medicine, Samsung Medical Center, Sungkyunkwan University School of Medicine, Seoul, Republic of Korea

**Keywords:** Febrile neutropenia, Pneumonia, Cancer, Combination antibiotic regimens

## Abstract

**Background:**

Although β-lactam monotherapy may be sufficient in non-critically ill patients with community-acquired pneumonia, the value of combination antibiotic regimens in community-onset neutropenic pneumonia remains unclear.

**Methods:**

A retrospective cohort study was conducted to compare the effects of combination antibiotic regimens to those of β-lactam monotherapy in cancer patients with community-onset neutropenic pneumonia. Electronic medical records of patients diagnosed with community-onset neutropenic pneumonia between March 1995 and February 2015 at a tertiary care center were reviewed.

**Results:**

During the study period, 165 cancer patients with community-onset neutropenic pneumonia were identified. Seventy-two patients received β-lactam monotherapy and 93 received combination therapy (β-lactam plus either a macrolide or fluoroquinolone). Causative pathogens were identified in 27.9% of the patients, and only two were positive for atypical pathogens. Although 30-day mortality was higher in the β-lactam group (15.3% versus 4.3%; *P* = 0.015), combination therapy was not associated with a statistically significant survival benefit in the multivariate analysis (hazard ratio 0.85, 95% confidence interval 0.20–3.67; *P* = 0.827). Duration of neutropenia, C-reactive protein level, and Multinational Association for Supportive Care in Cancer risk index were significant factors for 30-day mortality. In a subgroup analysis of patients treated with cefepime, the most frequently used β-lactam (63.0%), combination therapy also showed no significant survival benefit.

**Conclusions:**

Combination antibiotic regimens were not associated with a survival benefit over β-lactam monotherapy in the treatment of community-onset neutropenic pneumonia. Unnecessary combination therapy should be reconsidered in cancer patients who are at high risk for adverse drug reactions and colonization with multi-drug resistant organisms.

**Electronic supplementary material:**

The online version of this article (10.1186/s41479-019-0061-1) contains supplementary material, which is available to authorized users.

## Background

Febrile neutropenia, an inevitable side effect of chemotherapy, contributes to high mortality among cancer patients [1]. Among various clinical presentations of febrile neutropenia, pneumonia is considered high-risk [2]. Guidelines do not specify empirical antibiotics for the treatment of neutropenic pneumonia, i.e. community-onset pneumonia with febrile neutropenia. Although the European Society for Medical Oncology guidelines recommend coverage of atypical pathogens by adding macrolide antibiotics in managing neutropenic pneumonia, the level of evidence and grade of recommendation are very low [3]. Most clinicians choose a combination of an antipseudomonal β-lactam with azithromycin or fluoroquinolone targeting neutropenic fever and community-acquired pneumonia (CAP). However, the indiscriminate use of macrolide or fluoroquinolone in cancer patients receiving chemotherapy may result in unexpected side effects such as cardiovascular events and acquisition of resistance [4–7]. This study aimed to answer the question of whether combination therapy is necessary in patients with neutropenic pneumonia.

In the case of CAP, there has been already controversy about whether to start combination therapy as empirical antibiotics. The addition of macrolide or fluoroquinolone is based on the treatment for an atypical pathogen, antibiotic synergism, and anti-inflammatory effect. However, several studies have shown that β-lactam monotherapy is non-inferior to combination therapy [8–10]. Some have suggested that monotherapy may be considered in patients with low-risk CAP [11, 12]. Likewise, questions may arise as to whether combination therapy should be considered when neutropenic pneumonia is diagnosed in cancer patients who are receiving chemotherapy but with good performance status. To further investigate this question, we compared the effects of combination antibiotic regimens to those of β-lactam monotherapy in cancer patients with community-onset neutropenic pneumonia who were managed at a tertiary care center during a 20-year period.

## Methods

### Study design and population

A retrospective cohort study was conducted to compare the effects of combination antibiotic regimens to those of β-lactam monotherapy for the treatment of community-onset pneumonia in neutropenic cancer patients. We reviewed the electronic medical records of individuals who were admitted and diagnosed with pneumonia between March 1995 and February 2015 at Samsung Medical Center, a 1950-bed tertiary care university hospital in Seoul, Republic of Korea. Adult (age ≥ 18 years) cancer patients who were diagnosed with community-onset neutropenic pneumonia during the study period were included in the analysis. We exclusively included chemotherapy-induced neutropenia in the present study, and underlying cancers included solid cancers, lymphoma, and multiple myeloma. Hematologic malignancies such as acute leukemia, aplastic anemia, or myelodysplastic syndrome that accompany prolonged neutropenia due to intensive chemotherapy or the nature of the disease, were excluded from the analysis because prolonged neutropenia, a strong prognostic factor of febrile neutropenia, may obscure the effect of antibiotics [1, 13, 14]. Patients with lymphoma and multiple myeloma who underwent bone marrow transplantation were also excluded for the same reason. The study population was divided into two groups according to antibiotic regimen. Patients who were re-admitted 7 days after discharge were excluded because they were assumed to have hospital-acquired pneumonia. The combination group included patients who were treated with a combination of a β-lactam and a macrolide or fluoroquinolone, and the β-lactam group included patients who received β-lactam therapy only. Additional use of other classes of antibiotics that do not have activity for atypical bacterial pathogens, such as aminoglycosides or vancomycin, was not considered when classifying study groups. The primary outcome was 30-day all-cause mortality, and secondary outcomes were 90-day all-cause mortality, length of hospital stay, and complications. The study protocol was approved by the Institutional Review Board of Samsung Medical Center.

### Data collection

Retrospectively collected data included demographics, underlying diseases, clinical presentations and outcomes, laboratory test results, types and duration of antibiotic therapy, and microbiologic data. Microbiologic data included cultures of blood and respiratory specimens, pneumococcal and *Legionella* urinary antigen, influenza antigen, and mycoplasma antibody. The severity of neutropenic pneumonia was estimated using the CURB-65 score and the Multinational Association for Supportive Care in Cancer (MASCC) risk index [15, 16].

### Definitions

Pneumonia was defined as the presence of parenchymal infiltration on chest radiography (newly appeared infiltration or aggravation of preexisting lesions) with relevant respiratory symptoms, including cough, sputum production, shortness of breath, or pleuritic chest pain [17]. Pneumonia was considered to be of community-onset if it developed in the community or within 48 h after hospital admission [17, 18]. Neutropenia was defined as an absolute neutrophil count < 500 cells/mm^3^ or an absolute neutrophil count expected to decrease to < 500 cells/mm^3^ during the next 48 h [2]. Fever was defined as body temperature ≥ 38.0 °C. The response to chemotherapy was evaluated according to the World Health Organization criteria or Response Evaluation Criteria in Solid Tumors guidelines [19–21]. The mycoplasma antibody test was considered positive if a single titer was highly elevated (≥1:320) or a four-fold increase in the titer was detected in a follow-up sample [22].

### Statistical analyses

The sample size calculation for the study resulted in a sample size of 134 patients who were to be in a 1:1 allocation ratio to achieve a power of 95%. To compare the two groups, the Pearson χ^2^ test and Fisher’s exact test were used for categorical variables, and Student’s *t*-test and the Mann-Whitney U test for continuous variables. The Cox proportional hazard model was used to examine the associations of the antibiotic regimens with 30- and 90-day mortality in neutropenic pneumonia after adjusting for potential confounding factors. All collected variables with any relevance to outcomes were evaluated by univariate analysis, and those with statistical significance were included in the multivariate analysis. All *P*-values were two-tailed, and those < 0.05 were considered to be statistically significant. All statistical analyses were performed using SPSS Statistics version 20.0 for Windows (IBM Corp., Armonk, NY, USA).

## Results

### Baseline characteristics, antibiotic types, severity, and cancer status of neutropenic pneumonia

During the study period, 165 cancer patients with community-onset neutropenic pneumonia were identified: 72 were classified in the β-lactam group and 93 in the combination group. Blood cultures were performed in 100% of the enrolled patients and cultures of lower respiratory specimens in 99.0% of the patients. Causative pathogens of neutropenic pneumonia were identified in 27.9% of the patients, of which *Streptococcus pneumoniae* was the most common followed by *Pseudomonas aeruginosa*, *Staphylococcus aureus*, and *Klebsiella pneumoniae* (23.9, 19.6, 15.2, and 13.0%, respectively; Additional file 1: Table S1). Serologic tests for *Mycoplasma pneumoniae* were conducted in 20 patients (12.1%), two of which yielded positive results. The urinary antigen tests for legionella were performed in 75 patients (45.5%), and all showed results. No patient received oral prophylactic antibiotics before the onset of neutropenic fever.

The baseline characteristics, types of antibiotics, and the severity of neutropenic pneumonia are presented in Table 1. Baseline characteristics were similar between the two groups, except for male predominance in the β-lactam group and a higher prevalence of chronic renal disease in the combination group. Ceftazidime was used more frequently in the β-lactam group, while piperacillin-tazobactam was used more in the combination group. Ceftazidime was administered with tobramycin for an initial 3–5 days, and 23.6% of the β-lactam group and 19.4% of the combination group used an initial vancomycin combination (*P* = 0.507). In the combination group, azithromycin was most frequently used for coverage of atypical pathogens, followed by levofloxacin, ciprofloxacin, and moxifloxacin (75.3, 11.8, 8.6, and 4.3%, respectively). The duration of antibiotic administration was similar between the two groups. Severity variables, including the presence of bacteremia, duration of neutropenia, C-reactive protein (CRP) level, CURB-65 score, and MASCC risk index were not statistically different between the two groups (all *P* > 0.05).

**Table 1:** Baseline characteristics, types of β-lactams, and severity of patients with neutropenic pneumonia

Variables	β-lactam group (*n* = 72)	Combination group (*n* = 93)	*P*-value
Demographic data			
Age, years	61 ± 11	62 ± 10	0.296
Male sex	59 (81.9%)	59 (63.4%)	0.009
Smoking history	29 (40.3%)	27 (29.0%)	0.130
Comorbid conditions			
Hypertension	14 (19.4%)	28 (30.1%)	0.119
Diabetes mellitus	5 (6.9%)	12 (12.9%)	0.212
Cardiovascular diseases	5 (6.9%)	2 (2.2%)	0.130
Chronic lung diseases	3 (4.2%)	6 (6.5%)	0.733
Chronic liver diseases	3 (4.2%)	3 (3.2%)	1.000
Chronic renal diseases	0 (0.0%)	6 (6.5%)	0.036
Types of β-lactams			
Cefepime	40 (55.6%)	64 (68.8%)	0.080
Ceftazidime^a^	20 (27.8%)	2 (2.2%)	< 0.001
Piperacillin/tazobactam	6 (8.3%)	21 (22.6%)	0.014
Carbapenem	6 (8.3%)	6 (6.5%)	0.644
Duration of antibiotic administration, days	12.4 (8.9–16.2)	11.7 (9.1–14.4)	0.646
Severity variables			
Presence of bacteremia	16 (22.0%)	12 (12.9%)	0.114
Duration of neutropenia, days	1.5 (0.9–3.1)	1.4 (1.0–2.9)	0.773
CRP, mg/dL	15.9 (6.5–24.0)	17.0 (6.4–25.9)	0.669
CURB-65	1 (0–2)	1 (0–2)	0.898
MASCC risk index	16 (13–18)	16 (16–21)	0.059

Data are expressed as number (%) of patients, mean ± SD, or median (IQR)

^a^Ceftazidime was administered with tobramycin for an initial 3–5 days

Abbreviations: *CRP* C-reactive protein, *MASCC* Multinational Association for Supportive Care in Cancer, *SD* Standard deviation, *IQR* interquartile range

Types and status of cancers in patients with neutropenic pneumonia were not differently distributed between the two groups (Table 2, all *P* > 0.05). Lung cancer was the most common type of cancer, followed by lymphoma and breast cancer (44.8, 14.6, and 11.5%, respectively). Sixty-seven patients (40.6%) received chemotherapy for curative purposes, while the remainders (59.4%) were in palliative settings. Among patients in palliative settings, more than half (54.1%) experienced progression or relapse of underlying cancers despite previous chemotherapy.

**Table 2:** Types and status of cancers in patients with neutropenic pneumonia

Variables	β-lactam group (*n* = 72)	Combination group (*n* = 93)	*P*-value
Type of cancer			
Head and neck	3 (4.2%)	1 (1.1%)	0.319
Lung	36 (50.0%)	38 (40.9%)	0.242
Breast	7 (9.7%)	12 (12.9%)	0.526
Esophageal and gastric	8 (11.1%)	6 (6.5%)	0.287
Colorectal	1 (1.4%)	5 (5.4%)	0.233
Hepatobiliary and pancreatic	0 (0.0%)	3 (3.2%)	0.258
Bladder	1 (1.4%)	5 (5.4%)	0.233
Prostate	0 (0.0%)	4 (4.3%)	0.133
Melanoma and sarcoma	1 (1.4%)	5 (5.4%)	0.233
Lymphoma and myeloma	15 (20.8%)	13 (14.0%)	0.245
Cancer status			
Curative setting	32 (44.4%)	35 (37.6%)	0.377
Neoadjuvant and adjuvant	12 (16.7%)	20 (21.5%)	0.436
Definitive	20 (27.8%)	15 (16.1%)	0.069
Palliative setting^a^	40 (55.6%)	58 (62.4%)	0.377
Complete response	1 (2.5%)	0 (0.0%)	0.436
Partial response	2 (5.0%)	4 (6.9%)	0.697
Stable disease	0 (0.0%)	4 (6.9%)	0.133
Progressed or relapse	22 (55%)	31 (53.4%)	0.705
Before evaluation	15 (37.5%)	19 (32.8%)	0.949

Data are expressed as number (%) of patients

^a^The response to chemotherapy was evaluated according to the World Health Organization (WHO) criteria or Response Evaluation Criteria in Solid Tumors (RECIST) guidelines

### Outcomes and complications of patients with neutropenic pneumonia

The outcomes and complications of patients with neutropenic pneumonia are summarized in Table 3. In the β-lactam group, 30-day (15.3% versus 4.3%, *P* = 0.015) and 90-day all-cause mortality (20.8% versus 7.5%, *P* = 0.013) were significantly higher. Length of hospital stay, pneumonia recurrence, and complications and adverse effects, including complicated pleural effusion, *Clostridium difficile*-associated colitis, and cardiac events, were similar between the two groups (all *P* > 0.05).

**Table 3:** Outcomes and complications of patients with neutropenic pneumonia

Variables	β-lactam group (*n* = 72)	Combination group (*n* = 93)	*P*-value
30-day all-cause mortality	11 (15.3%)	4 (4.3%)	0.015
90-day all-cause mortality	15 (20.8%)	7 (7.5%)	0.013
Length of hospital stay, days	5.7 (4.4–10.9)	6.0 (4.3–8.6)	0.632
Recurrence of pneumonia^a^	4 (5.6%)	3 (3.2%)	0.700
Complications and adverse effects^a^			
Complicated pleural effusion	1 (1.4%)	2 (2.2%)	1.000
CDAD	3 (4.2%)	0 (0.0%)	0.081
Cardiac events^b^	4 (5.6%)	4 (4.3%)	0.730
Others^c^	8 (11.4%)	7 (7.5%)	0.394

Data are expressed as number (%) of patients or median (IQR)

^a^Within 90 days

^b^Cardiac events included arrhythmia, heart failure, and myocardial infarction

^c^Others included diarrhea, gastrointestinal bleeding, pneumothorax, epilepsy, and back pain

Abbreviations: *CDAD Clostridium difficile*-associated diarrhea, *IQR* interquartile range

To identify potential confounding factors of 30-day all-cause mortality, all relevant variables were evaluated in a univariate fashion using the Cox proportional hazard model (Table 4). Underlying lung cancer, palliative setting, neutropenia duration, CRP level, MASCC risk index, combination therapy, and the usage of cefepime and carbapenem were statistically significant factors of 30-day all-cause mortality in the univariate analysis. In the multivariate analysis of these variables, combination therapy did not maintain a significant association (hazard ratio [HR] 0.85, 95% confidence interval [CI] 0.20–3.67; *P* = 0.827). Duration of neutropenia (HR 1.27, 95% CI 1.05–1.53; *P* = 0.013), CRP level (HR 1.06, 95% CI 1.00–1.11; *P* = 0.021), and MASCC risk index (HR 0.80, 95% CI 0.65–0.99; *P* = 0.042) were persistent statistically significant factors of 30-day all-cause mortality. A multivariate analysis for 90-day all-cause mortality was performed (Additional file 2: Table S2). Combination therapy was not significantly associated with 90-day all-cause mortality (HR 0.67, 95% CI 0.23–2.76; *P* = 0.449), while palliative setting of chemotherapy (HR 12.81, 95% CI 1.78–92.20; *P* = 0.011), duration of neutropenia (HR 1.16, 95% CI 1.07–1.25; *P* = < 0.001), CRP level (HR 1.05, 95% CI 1.01–1.09; *P* = 0.020), and MASCC index (HR 0.84, 95% CI 0.72–0.98; *P* = 0.024) were statistically related to 90-day all-cause mortality.

**Table 4:** Multivariate analysis of 30-day all-cause mortality due to neutropenic pneumonia

Variables	Univariate analysis		Multivariate analysis	
	HR (95% CI)	*P*-value	HR (95% CI)	*P*-value
Lung cancer	3.55 (1.13–11.15)	0.030	1.60 (0.30–8.63)	0.586
Palliative setting	4.83 (1.09–21.43)	0.038	58.61 (0.26–13,356.90)	0.142
Duration of neutropenia	1.08 (1.03–1.15)	0.003	1.27 (1.05–1.53)	0.013
CRP	1.04 (1.00–1.08)	0.035	1.06 (1.00–1.11)	0.021
MASCC risk index	0.80 (0.68–0.95)	0.010	0.80 (0.65–0.99)	0.042
Combination therapy	0.27 (0.09–0.85)	0.025	0.85 (0.20–3.67)	0.827
Types of β-lactams				
Cefepime	0.28 (0.10–0.81)	0.019	0.64 (0.17–2.46)	0.518
Ceftazidime	2.05 (0.58–7.25)	0.268		
Piperacillin-tazobactam	1.42 (0.40–5.02)	0.589		
Carbapenem	5.95 (1.89–18.70)	0.002	0.28 (0.01–50.00)	0.631

Abbreviations: *CI* confidence interval, *CRP* C-reactive protein, *HR* hazard ratio, *MASCC* Multinational Association for Supportive Care in Cancer

### Subgroup analysis of cefepime-treated patients with neutropenic pneumonia

Although we performed a multivariate analysis to adjust for potential confounding factors, the types of β-lactams were significantly unbalanced between the two groups. To verify the effect of combination therapy on all-cause mortality due to neutropenic pneumonia, we also performed a subgroup analysis controlling for β-lactam antibiotics, specifically cefepime which made up the highest proportion among the various β-lactams (63.0%). The same variables used in the analysis of the original cohort were included in the multivariate analyses for 30- and 90-day all-cause mortality. In the multivariate analyses of the cefepime-treated subgroup, combination therapy was not significantly associated with either 30-day (HR 0.76, 95% CI 0.06–9.18; *P* = 0.965) or 90-day all-cause mortality (HR 0.78, 95% CI 0.18–3.45; *P* = 0.741).

## Discussion

This study showed that neither monotherapy nor combination therapy was associated with 30- and 90-day mortality in the treatment of neutropenic pneumonia. Although 30-day and 90-day all-cause mortality were higher in the β-lactam group than in the combination group before adjustment, this finding suggests that more patients in the β-lactam group had confounding factors for poor prognosis, including lung cancer, longer duration of neutropenia, or bacteremia, than patients in the combination group.

To our knowledge, this is the first study to compare the clinical efficacy of monotherapy and combination therapy in the treatment of neutropenic pneumonia, extending the concept of CAP. It has been already argued that monotherapy is sufficient for mild cases of CAP without neutropenia [11, 12]. The reasons for combination treatment is to cover atypical pathogens, antibiotics synergism, or anti-inflammatory effects. However, in fact, the proportion of atypical pathogens is known to be low in non-critically ill patients with CAP [8, 23]. A lack of synergistic effect between macrolide and β-lactam against *S. pneumoniae* has been described [24–26]. To date, no studies have shown the direct association between combination therapy and anti-inflammatory effect. Several studies have proven that β-lactam monotherapy is non-inferior to combination treatment [8–10, 26].

The concept that combination treatment may be unnecessary can be applied to neutropenic pneumonia. Cancer patients are at high risk for adverse drug reactions such as gastrointestinal and cardiogenic toxicity. In addition, cancer patients are more likely to be colonized with multi-drug resistant organisms with exposure to antibiotics. These problems can be overcome without the use of unnecessary antibiotics. This study showed that the rate of atypical pathogens was low although not all patients were examined. β-lactam monotherapy was not associated with 30- and 90-day mortality in the multivariate analysis. Rather, predictable variables such as duration of neutropenia, CRP level, and MASCC risk index were associated with mortality in neutropenic pneumonia. This result may support the fact that monotherapy is sufficient in non-critically ill patients with neutropenic pneumonia.

Due to the extended study period of 20 years, there were considerable differences in the types of β-lactams between the two groups. The preferred regimen for neutropenic pneumonia at our center was changed from ceftazidime combined with tobramycin to cefepime after 2002 due to increasing ceftazidime-resistance [27]. Instead of a tobramycin combination, clinicians tended to select either a macrolide or fluoroquinolone combinations to cover atypical pathogens in neutropenic pneumonia. As these changes in preferred regimens depending on the period may have introduced some bias, we performed a subgroup analysis of cefepime-treated patients, and also found no difference in outcomes between the two groups, supporting the results of the main cohort.

There are several limitations to our study. First, the 20-year study period may lead to heterogeneity in the study population. However, we inevitably expanded the study period to obtain statistical significance in comparing the two groups. We conducted both multivariate analyses to adjust for potential confounding factors and subgroup analysis of cefepime-treated patients to overcome this limitation. Second, as a limitation of retrospective studies, certain criteria have not been applied to the selection of the antibiotics, and the test results for diagnosis of pneumonia. Finally, screening for atypical pathogens was not performed in all patients and the *Legionella* test in this study covered only serotype 1. This is also the limitation of retrospective study, and efforts to find causative organisms of pneumonia through the tests including urinary antigen detection, respiratory virus PCR panels, sputum cultures are needed. Additional prospective studies are needed to modify this limitation.

In conclusion, this study revealed no differences in 30- and 90-day mortality between β-lactam monotherapy and combination therapy in cancer patients with non-severe community-onset neutropenic pneumonia. Monotherapy can reduce the chance of unwanted drug adverse reactions and acquisition of antibiotic resistance. Unnecessary combination therapy should be reconsidered in neutropenic pneumonia. Future studies are needed with better evaluations for atypical pathogens.

## Additional files

Additional file 1: **Table S1.** Causative pathogens of neutropenic pneumonia. (DOCX 15 kb)

Additional file 2: **Table S2.**. Multivariate analysis of 90-day all-cause mortality due to neutropenic pneumonia. (DOCX 15 kb)

## Abbreviations

CAP: Community-acquired pneumonia
CI: Confidence interval
CRP: C-reactive protein
HR: Hazard ratio
MASCC: Multinational association for supportive care in cancer
