# Supplementary material for: Treatment of community-onset pneumonia in neutropenic cancer patients: β-lactam monotherapy versus combination antibiotic regimens
Source: Pneumonia (Nathan). 2019 Jun 5;11:2. doi: 10.1186/s41479-019-0061-1 (PMC6549334; doi:10.1186/s41479-019-0061-1)
Supplement: Supplementary file 1 — Table S1. Causative pathogens of neutropenic pneumonia. (DOCX 15 kb) [file 41479_2019_61_MOESM1_ESM.docx]

**Supplementary Table 1. Causative pathogens of neutropenic pneumonia**

| **Variables** | **β-lactam group**  (n = 72) | **Combination group**  (n = 93) | ***P*-value** |
| --- | --- | --- | --- |
| **Identification of causative pathogens** | 21 (29.2%) | 25 (26.9%) | 0.745 |
| *Streptococcus pneumoniae* | 4 (19.1%) | 7 (28.0%) |  |
| *Streptococcus mitis/oralis* | 2 (9.5%) | 1 (4.0%) |  |
| *Streptococcus pyogenes* | 1 (4.8%) | 0 (0.0%) |  |
| β-hemolytic streptococcus group G | 0 (0.0%) | 1 (4.0%) |  |
| *Staphylococcus aureus* | 1 (4.8%) | 6 (24.0%) |  |
| *Enterococcus faecium* | 2 (9.5%) | 0 (0.0%) |  |
| *Moraxella catarrhalis* | 0 (0.0%) | 1 (4.0%) |  |
| *Escherichia coli* | 2 (9.5%) | 0 (0.0%) |  |
| *Klebsiella pneumoniae* | 3 (14.8%) | 3 (12.0%) |  |
| *Serratia marcescens* | 0 (0.0%) | 1 (4.0%) |  |
| *Pseudomonas aeruginosa* | 5 (23.8%) | 4 (16.0%) |  |
| *Stenotrophomonas maltophilia* | 1 (4.8%) | 0 (0.0%) |  |
| *Acinetobacter baumannii* | 1 (4.8%) | 1 (4.0%) |  |
| *Mycoplasma pneumoniae*^a)^ | 1 (4.8%) | 1 (4.0%) |  |
| Influenza virus | 2 (9.5%) | 3 (12.0%) |  |

Data are expressed as number (%) of patients.

^a)^Mycoplasma antibody was only tested in 20 patients (6 in the β-lactam group and 14 in the combination group).
