# Supplementary material for: Treatment of community-onset pneumonia in neutropenic cancer patients: β-lactam monotherapy versus combination antibiotic regimens
Source: Pneumonia (Nathan). 2019 Jun 5;11:2. doi: 10.1186/s41479-019-0061-1 (PMC6549334; doi:10.1186/s41479-019-0061-1)
Supplement: Supplementary file 2 — Table S2.. Multivariate analysis of 90-day all-cause mortality due to neutropenic pneumonia. (DOCX 15 kb) [file 41479_2019_61_MOESM2_ESM.docx]

**Supplementary Table 2. Multivariate analysis of 90-day all-cause mortality due to neutropenic pneumonia**

| **Variables** | **Univariate analysis** | | | **Multivariate analysis** | |
| --- | --- | --- | --- | --- | --- |
|  | HR (95% CI) | *P*-value | HR (95% CI) | | *P*-value |
| **Lung cancer** | 3.61 (1.41-9.22) | 0.007 | 2.06 (0.66-6.48) | | 0.215 |
| **Palliative setting** | 5.03 (1.49-17.00) | 0.009 | 12.81 (1.78-92.20) | | 0.011 |
| **Duration of neutropenia** | 1.08 (1.02-1.14) | 0.008 | 1.16 (1.07-1.25) | | <0.001 |
| **CRP** | 1.04 (1.01-1.07) | 0.022 | 1.05 (1.01-1.09) | | 0.020 |
| **MASCC index** | 0.82 (0.72-0.94) | 0.004 | 0.84 (0.72-0.98) | | 0.024 |
| **Presence of bacteremia** | 2.70 (1.10-6.63) | 0.030 | 0.85 (0.26-2.76) | | 0.785 |
| **Combination therapy** | 0.34 (0.14-0.84) | 0.019 | 0.67 (0.23-2.76) | | 0.449 |
| **Types of β-lactams** |  |  |  | |  |
| Cefepime | 0.44 (0.19-1.01) | 0.053 |  | |  |
| Ceftazidime | 2.54 (0.94-6.89) | 0.067 |  | |  |
| Piperacillin/tazobactam | 0.93 (0.27-3.14) | 0.903 |  | |  |
| Carbapenem | 3.90 (1.32-11.55) | 0.014 | 0.29 (0.01-11.18) | | 0.507 |

Abbreviations: CI, confidence interval; CRP, C-reactive protein; HR, hazard ratio; MASCC, Multinational Association for Supportive Care in Cancer
